# Aphid Nymphs Experiencing Diurnal Temperature Fluctuation Alter the Toxicity of Adults Depending on the Role of the Insecticide Temperature Coefficients

**DOI:** 10.3390/biology14111543

**Published:** 2025-11-03

**Authors:** Biao Liu, Bo Gao, Xu Cheng, Yun-Wei Liu, Kun Xing, Fei Zhao

**Affiliations:** Shanxi Key Laboratory of Integrated Pest Management in Agriculture, College of Plant Protection, Shanxi Agricultural University, Taiyuan 030031, China; liubiao@sxau.edu.cn (B.L.); 20232356@stu.sxau.edu.cn (B.G.); 202420294@stu.sxau.edu.cn (X.C.); 202430477@stu.sxau.edu.cn (Y.-W.L.)

**Keywords:** climate change, delay effect, insecticide, temperature amplitude, temperature coefficient, wheat aphid

## Abstract

**Simple Summary:**

As environmental stressors, climate change and insecticides interact in intricate ways that can adversely affect the growth and reproductive success of living organisms. This study explores how temperature amplitudes experienced during the nymphal stage affect the insecticide sensitivity of the adult *Sitobion avenae* (Fabricius) (Hemiptera: Aphididae). Using two insecticides with opposing temperature coefficients of imidacloprid (positive) and beta-cypermethrin (negative), we found that temperature amplitude significantly altered the survival, longevity, and reproduction of the adult. Notably, both insecticides mitigated or even reversed the negative effects of wide temperature amplitudes. These findings highlight the importance of considering diurnal temperature fluctuations and insecticide temperature coefficients when assessing pesticide risks under climate change.

**Abstract:**

Climate change and insecticides negatively impact organism development and reproduction. Previous studies on climate change have focused on average temperature while ignoring diurnal temperature fluctuations. Therefore, this study investigates the interaction effects of the nymph experiencing temperature amplitudes (TAs) (+/−0, +/−6, +/−12 °C) at the same average temperature (22 °C) and two insecticides (beta-cypermethrin: negative temperature coefficient NT, imidacloprid: positive temperature coefficient PT) in the adult phenotypes and population parameter of *S. avenae*. The findings revealed that wide amplitude (+/−12 °C) significantly decreased fecundity, daily nymph reproduction, and the intrinsic rate of increase, while it significantly enhanced early fecundity. Medium amplitude (+/−6 °C) significantly lowered the intrinsic rate of increase. Notably, insecticides mitigated or even reversed the harmful impact of wide amplitude on adults. Under PT treatment, longevity was significantly higher than that under 22 °C. Under NT treatment, survival was noticeably greater than that under 22 °C. The interaction between insecticide and medium amplitude positively influenced adult phenotypes, with both PT and NT treatments resulting in higher survival, longevity, fecundity, and daily nymph production compared to 22 °C. These findings support the theory of climate-induced poison sensitivity and indicate that insecticide temperature coefficient is crucial in assessing insecticide safety risks under climate change scenarios.

## 1. Introduction

The intensification of climate change and the widespread use of insecticides pose considerable threats to the development and fecundity of organisms on earth and can even result in catastrophic harm [1,2]. Therefore, an in-depth understanding of the biological interactions between climate change and insecticides on organisms has emerged as a primary research focus in the fields of ecotoxicology and environmental research [3]. Previous studies have established three aspects of the interaction between insecticides and climate change. On the one hand, it is believed that the sensitivity of organisms to insecticides is restricted by environmental factors associated with climate change, that is, “climate-induced toxicant sensitivity (CITS)” [4,5]. Furthermore, exposure to chemical pollutants is thought to modify susceptibility to climate change, referred to as “toxicant-induced climate change sensitivity (TICS).” On the other hand, it is believed that the roles of CITS and TICS in biology cannot exist independently, although a close connection between the two exists [6]. Therefore, we require abundant experimental evidence to comprehensively understand the biological responses to the interaction between climate change and insecticides.

Most studies examining the effects of insecticides and global warming on insects exclusively focus on the effects of rising mean temperatures on insecticide toxicity [7] while largely disregarding the potential effects of diurnal temperature fluctuations. Diurnal temperature fluctuations and insecticides exert negative effects on insects; bifenthrin could increase the mortality of *Chironomus dilutus* (Shobanov, Kiknadze & Butler) [8], acetamiprid could decrease fecundity of *Frankliniella occidentalis* (Pergande) [9], and chlorpyrifos could decrease, heat tolerance, longevity, and anti-predation behavior of *Culex pipiens* (Linnaeus) [10,11]. However, they also exert positive effects on insects. The negative impact of chlorpyrifos on the adult survival of *C. pipiens* was less apparent under daily temperature variation than at the constant temperature [12]; temperature fluctuations and insecticides could reduce oxidative damage of *Lestes viridis* (Stephens) [13], enhance adult fecundity of *Nilaparvata lugens* (Stål) [14], and improve viral transmission by disease-carrying insects *Aedes aegypti* (Linnaeus) [15]. Furthermore, the interaction between insecticides and temperature fluctuations causes effects at the individual level and exerts considerable effects on higher-level biological structures, from populations to ecosystems. For example, *Daphnia magna* experiences substantial decreases in lifetime fecundity success and population growth rates when exposed to fluoxetine and temperature fluctuations [16], which can have broader implications for the entire aquatic ecosystem. Moreover, given the crucial role of *D. magna* in aquatic food webs [17], the effect of the interplay between climate warming and insecticides on insect populations may be underestimated.

In contrast to vertebrates, which undergo gradual growth and development, insects, being ectotherms with distinct life stages (i.e., different developmental stages), have the ability to decouple thermal conditions and physiological responses from one life stage to the next [18]. Moreover, different developmental stages of insects may reveal different physiological and ecological responses to environmental stress, implying that insects at different life stages may not respond consistently to the interaction between diurnal temperature fluctuations and insecticides [19]. For example, when *Gryllus lineaticeps* (Stål) experience warming and insecticide exposure during nymphal development, their survival, adult body weight, and size are reduced. In contrast, warming and insecticide exposure during the adult stage reduce their longevity and growth rate. Both nymphs and adults are considerably affected by warming, whereas the adult stage is more sensitive to insecticide exposure [20]. Previous studies have shown that when adults *S. avenae* experience wide temperature fluctuation and imidacloprid, their longevity, fecundity, and survival are significantly reduced. When exposed to the wide temperature fluctuation and beta-cypermethrin, the intrinsic rate of increase is also reduced [21]. However, when generations of *S. avenae* experience wide temperature fluctuation and imidacloprid, their longevity, fecundity, survival, intrinsic rate of increase, and net reproductive rate are significantly reduced. In contrast, when exposed to wide temperature fluctuation and beta-cypermethrin, the survival, longevity, fecundity, intrinsic rate of increase, generation time, and net reproductive rate were all higher than those under constant temperature, and a significant promoting effect was exhibited [22]. Therefore, when investigating the effects of the interplay between diurnal temperature fluctuations and insecticides on insects, it is essential to account for the potentially varied responses to stress that insects may exhibit at different developmental stages.

*S. avenae* is a highly prevalent and extremely damaging agricultural pest [23]. Multiple generations of this species occur within a single year, and it is characterized by large fecundity numbers, small individuals, and high sensitivity to environmental stress. *S. avenae* serves as an excellent model for examining the effects of environmental changes on pests. Currently, the primary insecticides used for controlling *S. avenae* in the field are imidacloprid, characterized by a positive temperature (PT) coefficient, and beta-cypermethrin, which exhibits a negative temperature (NT) coefficient [24]. Research has shown that temperature amplitude significantly affects the longevity, survival, development, population parameters, fecundity, and temperature tolerance of *S. avenae* [25,26]. The reaction of *S. avenae* to insecticides across temperature variations has significant implications for insecticide application amid climate change and the safety assessment of insecticides.

Therefore, we investigated the interaction effects of insecticides with different temperature coefficients (beta-cypermethrin NT and imidacloprid PT) and nymphs experiencing temperature amplitudes (22 +/− 0, 22 +/− 6, and 22 +/− 12 °C) on the traits of adults *S. avenae*, such as longevity, survival, early fecundity, overall fecundity, intrinsic rate of increase, and daily nymph production. This study aimed to address the following questions: (1) Will the interaction between different insecticides and temperature amplitudes affect the life history traits and the population parameters of *S. avenae*? (2) Will this interaction vary based on the different temperature coefficients? (3) What is the significance of this interaction for biological risk assessments under climate change conditions and integrated pest management strategies?

## 2. Materials and Methods

### 2.1. Insects

In May 2016, *S. avenae* was collected from the winter wheat experimental field of the Shanxi Academy of Agricultural Sciences (116°16′ N, 35°55′ E). At that time, the winter wheat was in the heading-to-filling stage, and the *S. avenae* population was also in its peak occurrence period; a total of 500 aphids were collected and brought back for rearing. *S. avenae* specimens were collected in accordance with local regulations and permits. No protected areas or endangered species were involved. They were raised on winter wheat plants (15–20 cm) and placed in breathable, transparent insect-rearing cages measuring 60 cm × 60 cm × 60 cm. These cages were kept in an insect-rearing room with a relative humidity of 40–60%, a temperature of 22 +/− 1 °C, and a photoperiod of 16 h light and 8 h dark. The *S. avenae* population had been reared indoors for a minimum of 9 years prior to the experiment. However, every other year in May, we collected supplemental field populations 3–4 times from the original collection site (approximately 500–600 *S. avenae* individuals each time) and reared them in a mixed manner with the existing laboratory population at a 1:1 mixing ratio, maintaining the total number of *S. avenae* under indoor rearing at around 2000. The purpose of this method is to maintain population genetic diversity and reduce the genetic drift caused by long-term indoor rearing.

### 2.2. Temperature Design

Analysis of meteorological data from wheat-producing regions in northern China in May 2024, the primary winter wheat-growing period, showed that daily diurnal temperature fluctuations approximately follow a sinusoidal temperature pattern (Figure S1A), with a mean daily temperature of 22 °C (Figure S1B) and a maximum diurnal temperature range of +/−12 °C (Figure S1C). The hourly relative humidity is basically between 40% and 60% every day from 21:00 to 05:00 the next day (Figure S2A), and there were 18 days accounting, for 58.06% of May’s total days, where the relative humidity ranged between 40% and 60%, excluding 2 days with concentrated rainfall (with respective daily mean relative humidity values of 92.4% and 83.3%) (Figure S2B). Consequently, we set up and simulated an average daily temperature of 22 °C and diurnal temperature variations of +/−0, +/−6, and +/−12 °C in an indoor setting (Figure S3A). A variable temperature design was developed in the artificial climate chambers, and a HOBO data logger (U23—001, Onset Computer, MA, USA) was employed to monitor the temperature in the artificial climate chambers in real-time at regular intervals of 20 min (Figure S3B). During the test, the artificial climate chambers maintained a photoperiod of 16 h of light (05:00–21:00) and 8 h of darkness (21:00–05:00). Relative humidity parameters were maintained between 40% and 60%. The laboratory temperature was maintained at roughly 22 °C using central air conditioning.

### 2.3. Insecticide Concentration

We selected imidacloprid (PT), beta-cypermethrin (NT), and solvent control (SC). These two insecticides are employed for year-round field control of aphids. Insecticide technical materials, i.e., imidacloprid (purity grade > 99%) and beta-cypermethrin (purity grade > 99%), were sourced from Yangzhou Suling Insecticide Chemical Co. Ltd. (Yangzhou, China). Two insecticides were used to treat wheat aphids at LC_10_ (lethal dose). First, five concentrations of imidacloprid (1.2, 3.6, 10.8, 32.4, and 97.2 μg/mL) and five concentrations of beta-cypermethrin (0.1, 0.5, 2.5, 12.5, and 62.5 μg/mL) were prepared with insecticide technical material and solvent, which is mixed with acetone (purity grade > 99%) and distilled water in a volume ratio of 1:1. The experimental control group was treated with pure solvent, which was a mixture of distilled water and acetone in a ratio of 1:1. Twelve hours before the insecticide treatment, we prepared insecticide solutions of different concentrations, which were then placed into small transparent glass bottles (5 cm in height × 2.5 cm in diameter) with airtight lids (rubber lids). These bottles were stored at room temperature (22 °C) in a light-proof environment for later use. This measure was to prevent insecticide volatilization and avoid changes in insecticide concentration. Second, each concentration treatment was conducted in four replicates, with 30 insects in each replicate. The virulence of 1-day-old *S. avenae* adults were assessed using the topical application method recommended by the Food and Agriculture Organization (FAO). In the experiment, we selected apterous aphids with roughly uniform body size. The capillary tube micro-topical applicator delivered a volume of 0.1 μL per aphid at a distance of 1–2 mm from the aphid body. We ensured that the droplet fell vertically to form a complete circular droplet (without spreading or rolling off) and landed on the aphid’s mesonotum. If the droplet fell off, the aphid escaped, or the application site was incorrect, the aphid was replaced and the application repeated; failed individuals were excluded from the sample size. After application, aphids were placed at room temperature (22 °C) for 3–4 min to stand until the solvent had completely evaporated; then, they were transferred to the insect rearing chamber to continue the experiment. Subsequently, the solvent control did not affect the survival of aphids, and the regression equation for toxicity was derived using Probit analysis in IBM SPSS Statistics 21 (SPSS Inc., Chicago, IL, USA). For beta-cypermethrin, the equation was y = −0.121 + 0.755 x, with *χ*^2^ = 1.128, df = 3, and *p* = 0.770. For imidacloprid, the equation was y = −0.817 + 1.011 x, with *χ*^2^ = 1.401, df = 3, and *p* = 0.705. Finally, the LC_10_ of imidacloprid (PT) was found to be 0.347 µg/mL (95% CI: 0.055 to 0.895), and the LC_10_ of beta-cypermethrin (NT) was found to be 0.029 µg/mL (95% CI: 0.003 to 0.096).

### 2.4. Test Procedure

**Figure 1:**
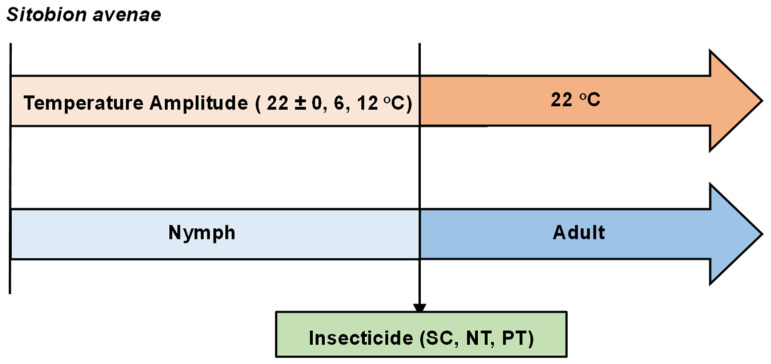
Experimental design encompassing the employed treatment methods.

We selected 432 newly nymphs (48 nymphs/treatment × 3 temperature amplitudes × 3 insecticide treatments) born within 4 h. To avoid the systematic errors caused by the operational errors of the climate chambers and the micro-environmental differences inside climate chambers, we placed one treatment with the same conditions in the same climate chamber. Each aphid was individually reared using a rearing tube (Chinese invention patent: A method for indoor rearing of aphids, ZL201710216994.4, China). This rearing tube consists of a transparent circular plastic tube, 7 cm in length × 2 cm in diameter. At the bottom of each rearing tube, there is a circular sponge block (2 cm in height × 2.5 cm in diameter) with a straight plant insertion slot centered on the top of the sponge block. The slot is 0.6 cm deep and 1.8 cm long, into which a 5 cm fresh wheat leaf is inserted. The top of the rearing tube is sealed with a circular sponge (2 cm in height × 2.5 cm in diameter). Afterwards, the individual rearing tubes are placed vertically in a rearing tray (20 cm in length × 15 cm in width × 4 cm in height), which is filled with 1.5–2 cm of wheat nutrient solution. Then, each aphid is exposed to different temperature amplitudes (22 +/− 0, 22 +/− 6, and 22 +/− 12 °C). When the *S. avenae* reached the 1-day-old adult stage, they were subjected to insecticide treatment (SC, PT, and NT) using the topical application method. Subsequently, all aphids were transferred to an insect rearing chamber for continued the experiment in the insect-rearing room (temperature: 22 °C; relative humidity: 40–60%; light–dark cycle: 16:8) (Figure 1). The death and fecundity of aphids were examined daily at 08:00. Any newly hatched or dead aphids were promptly removed until all tested aphids perished, thereby concluding the test. To ensure sufficient nutrition for *S. avenae*, the wheat leaves in the feeding tubes were replaced every 2 d.

### 2.5. Statistical Analysis

In the experiment, adult longevity represents the number of days from the age of 9 d until the death of an adult. After excluding individuals that escaped or died abnormally, the final number of individuals used for adult longevity and survival curves statistics was, in tota1, 345 (22 °C-SC: 41, 22 +/− 6 °C-SC: 44, 22 +/− 12 °C-SC: 38, and 22 °C-NT: 43, 22 +/− 6 °C-NT: 40, 22 +/− 12 °C-NT: 32, and 22 °C-PT: 38, 22 +/− 6 °C-PT: 41, 22 +/− 12 °C-PT: 28, respectively). Moreover, fecundity is defined as the total count of offspring produced from the age of 9 d until the adult’s death. The early fecundity represents the count of offspring produced in the first three days of maturity as a percentage of the total fecundity percentage. During the experiment, a small number of adults exhibited non-reproductive behavior. Therefore, in some treatments (22 +/− 12 °C-NT and 22 +/− 12 °C-PT), the number of individuals used to determine the fecundity and early reproductive rate statistics is smaller than that used for longevity statistics. The final number of individuals used for adult fecundity and early fecundity statistics was, in tota1, 335 (22 °C-SC: 41, 22 +/− 6 °C-SC: 44, 22 +/− 12 °C-SC: 38, and 22 °C-NT: 43, 22 +/− 6 °C-NT: 40, 22 +/− 12 °C-NT: 26, and 22 °C-PT: 38, 22 +/− 6 °C-PT: 41, 22 +/− 12 °C-PT: 24, respectively).

We used the Shapiro–Wilk test to evaluate the normality of the entire dataset and discovered that most of the data did not conform to a normal distribution. Therefore, to analyze the effects of insecticides, temperature amplitudes, and their interactions on adult fecundity and longevity, we employed generalized linear models with a gamma error distribution for a two-factor analysis and utilized Fisher’s least significant difference test for multiple comparisons among treatments. We employed the Cox proportional hazard model to analyze the effects of insecticides, temperature amplitudes, and their interactions on overall survival of adult insects. The survival curves of the adult specimens subjected to different treatments under a single factor were evaluated using the log-rank test method in conjunction with the Kaplan–Meier analysis. The Holm–Sidak test was used to make multiple comparisons between the two treatments. We randomly divided the daily fecundity of adults under each treatment into 3 replicates and used repeated measurements to analyze daily nymph reproduction. Data analysis was performed with IBM SPSS Statistics 21 (SPSS Inc., Chicago, IL, USA).

According to the method of Zhu [27], the calculation formula for the intrinsic rate of increase (rm) of wheat aphids was as follows: rm = ln (R0)/G, where “x” denotes the age of the aphid, “lx” represents the survival rate at various ages, and “mx” indicates the total fecundity at different ages. We used the bootstrap procedure method (parameter setting: rep = 99) in R version 4.3.2 to analyze the mean value and 95% confidence interval of each index of wheat aphid population parameters. The Kruskal–Wallis test was used for comparisons between different treatments.

## 3. Results

### 3.1. Effects of Temperature Amplitude and Insecticide on Survival

**Figure 2:**
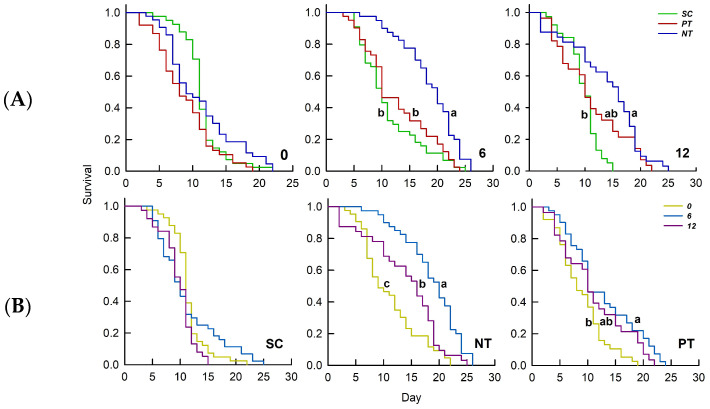
Effects of (**A**) temperature amplitude and (**B**) insecticide treatment experienced by *Sitobion avenae* nymphs on the survival curve. Different letters above the curve indicate significant differences between treatments (*p* < 0.05). The different temperature amplitudes (22 +/− 0, 22 +/− 6, and 22 +/− 12 °C) obtained are represented by 0, 6, and 12. SC, NT, and PT represent solvent control, beta-cypermethrin, and imidacloprid treatments, respectively.

Temperature amplitude did not significantly affect the survival (*χ*^2^ = 5.226, *n* = 345, *p* = 0.073). However, insecticide (*χ*^2^ = 14.027, *n* = 345, *p* < 0.001) and the interaction between temperature amplitude and insecticide (*χ*^2^ = 14.563, *n* = 345, *p* = 0.006) had significant impacts on survival (Table 1). At +/−0 °C, insecticides did not significantly influence survival (*χ*^2^ = 6.542, *n* = 122, *p* = 0.083). Conversely, at +/−6 °C and +/−12 °C, insecticides significantly affected survival (*χ*^2^ = 26.212, *n* = 125, *p* < 0.001; *χ*^2^ = 16.936, *n* = 98, *p* < 0.001), with higher survival rates following NT treatment compared to PT and SC treatments. Under SC treatment, temperature amplitude did not significantly impact survival (*χ*^2^ = 4.617, *n* = 123, *p* = 0.099). In contrast, survival rates under PT and NT treatments were significantly influenced by temperature amplitude (*χ*^2^ = 31.878, *n* = 107, *p* < 0.001; *χ*^2^ = 11.710, *n* = 115, *p* = 0.003). The survival rates were elevated at +/−6 °C compared to those observed at +/−0 °C and +/−12 °C (Figure 2).

### 3.2. Effects of Temperature Amplitude and Insecticide on Longevity

**Figure 3:**
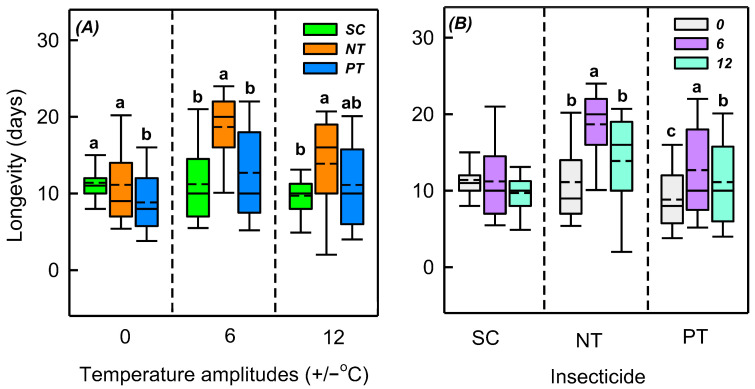
Box plot of effects of (**A**) temperature amplitude and (**B**) insecticide treatment experienced by *Sitobion avenae* nymphs on longevity. The upper and lower boundaries of the box indicate the 75th and 25th percentiles of the dataset, respectively. The black solid line and short dashed line within the box represent the median and mean values, respectively. Error bars represent the standard deviation. Different letters above each box indicate significant differences (*p* < 0.05) among the treatments. The different temperature amplitudes (22 +/− 0, 22 +/− 6, and 22 +/− 12 °C) obtained are represented by 0, 6, and 12. SC, NT, and PT represent solvent control, beta-cypermethrin, and imidacloprid treatments, respectively.

Temperature amplitude (*χ*^2^ = 36.353, *n* = 345, *p* < 0.001), insecticide (*χ*^2^ = 42.078, *n* = 345, *p* < 0.001), and their interaction (*χ*^2^ = 26.739, *n* = 345, *p* < 0.001) had a significantly affected on the longevity (Table 2). Insecticides notably influenced longevity at +/−0, +/−6, and +/−12 °C (*χ*^2^ = 8.792, *n* = 122, *p* = 0.012; *χ*^2^ = 30.692, *n* = 125, *p* < 0.001; *χ*^2^ = 9.061, *n* = 98, *p* = 0.011). At +/−0, +/−6, and +/−12 °C, the differences in longevity between NT and SC treatments were −0.3, 7.5, and 4.2 d, respectively, and the differences in longevity between PT and SC treatments were −2.6, 1.5, and 1.4 d, respectively. Under the SC treatment, the temperature amplitude did not significantly affect the longevity (*χ*^2^ = 5.031, *n* = 123, *p* = 0.081). However, under both PT and NT treatments, the temperature amplitude significantly influenced the longevity (*χ*^2^ = 6.837, *n* = 107, *p* = 0.033; *χ*^2^ = 29.880, *n* = 115, *p* < 0.001). Under the NT treatment, compared with +/−0 °C, the longevity at +/−6 °C and +/−12 °C increased by 7.6 and 2.8 d, respectively. Under the PT treatment, compared with +/−0 °C, the longevity of +/−6 °C and +/−12 °C increased by 3.9 and 2.3 d, respectively (Table S2 and Figure 3).

### 3.3. Effects of Temperature Amplitude and Insecticide on Fecundity

**Figure 4:**
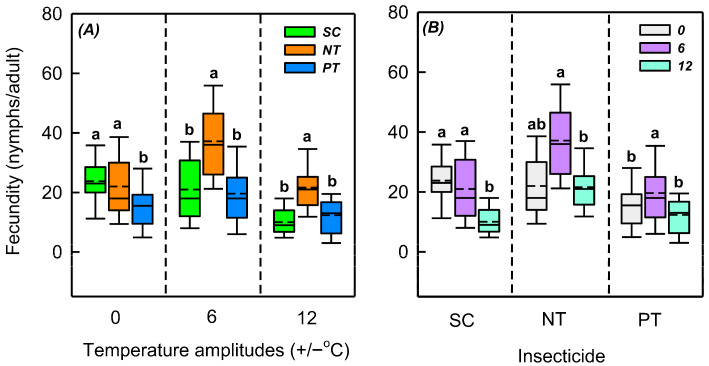
Box plot of effects of (**A**) temperature amplitude and (**B**) insecticide treatment experienced by *Sitobion avenae* nymphs on adult fecundity. The upper and lower boundaries of the box indicate the 75th and 25th percentiles of the dataset, respectively. The black solid line and short dashed line within the box represent the median and mean values, respectively. Error bars represent the standard deviation. Different letters above each box indicate significant differences (*p* < 0.05) among the treatments. The different temperature amplitudes (22 +/− 0, 22 +/− 6, and 22 +/− 12 °C) obtained are represented by 0, 6, and 12. SC, NT, and PT represent solvent control, beta-cypermethrin, and imidacloprid treatments, respectively.

Temperature amplitude (*χ*^2^ = 68.830, *n* = 335, *p* < 0.001), insecticide (*χ*^2^ = 74.899, *n* = 335, *p* < 0.001), and their interaction (*χ*^2^ = 42.621, *p* < 0.001) significantly influenced the fecundity (Table 2). At +/−0, +/−6, and +/−12 °C, insecticides markedly influenced the fecundity (*χ*^2^ = 17.352, *n* = 122, *p* < 0.001; *χ*^2^ = 37.161, *n* = 125, *p* < 0.001; *χ*^2^ = 31.139, *n* = 88, *p* < 0.001). At +/−0, +/−6, and +/−12 °C, the differences in fecundity between NT and SC treatments were −1.8, 16.2, and 11.6 nymphs/adult, respectively, and the differences in fecundity between PT and SC treatments were −8.3, −1.4, and 2.3 nymphs/adult, respectively. Under SC, PT, and NT treatments, the temperature amplitude significantly affected the fecundity (*χ*^2^ = 45.966, *n* = 123, *p* < 0.001; *χ*^2^ = 31.553, *n* = 103, *p* < 0.001; *χ*^2^ = 8.857, *n* = 109, *p* = 0.012). Under the SC treatment, compared with +/−0 °C, the fecundity of +/−6 °C and +/−12 °C decreased by 2.8 and 13.8 nymphs/adult, respectively. Under the NT treatment, compared with +/−0 °C, the fecundity at +/−6 °C increased by 15.2 nymphs/adult, and the fecundity at +/−12 °C reduced by 0.4 nymphs/adult. Under the PT treatment, compared with +/−0 °C, the fecundity at +/−6 °C increased by 4.1 nymphs/adult, and the fecundity at +/−12 °C decreased by 3.2 nymphs/adult (Table S2 and Figure 4).

### 3.4. Effects of Temperature Amplitude and Insecticide on Early Fecundity

**Figure 5:**
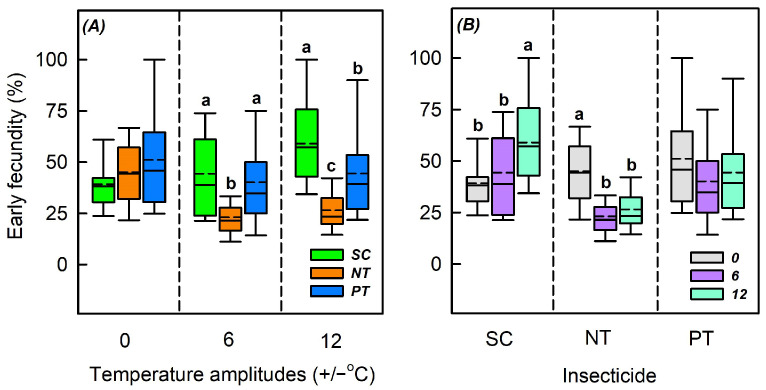
Box plot of effects of (**A**) temperature amplitude and (**B**) insecticide treatment experienced by S. avenae nymphs on adult early fecundity. The upper and lower boundaries of the box indicate the 75th and 25th percentiles of the dataset, respectively. The black solid line and short dashed line within the box represent the median and mean values, respectively. Error bars represent the standard deviation. Different letters above each box indicate significant differences (*p* < 0.05) among the treatments. The different temperature amplitudes (22 +/− 0, 22 +/− 6, and 22 +/− 12 °C) obtained are represented by 0, 6, and 12. SC, NT, and PT represent solvent control, beta-cypermethrin, and imidacloprid treatments, respectively.

Temperature amplitude (*χ*^2^ = 15.362, *n* = 335, *p* < 0.001), insecticide (*χ*^2^ = 42.405, *n* = 335, *p* < 0.001), and their interaction markedly influenced the early fecundity (*χ*^2^ = 40.831, *n* = 335, *p* < 0.001) (Table 2). At +/−0 °C, insecticides significantly affected the early fecundity (*χ*^2^ = 2.813, *n* = 122, *p* = 0.245). At +/−6 and +/−12 °C, insecticides markedly influenced the early fecundity (*χ*^2^ = 27.132, *n* = 125, *p* < 0.001; *χ*^2^ = 36.034, *n* = 88, *p* < 0.001). At +/−6 °C, the early fecundity lessened by 21.2% and 4.1% in the NT and PT treatments, respectively, compared to the SC treatment. At +/−12 °C, compared with the SC treatments, the early fecundity of NT and PT treatments reduced by 32.5% and 14.6%, respectively. Under both SC and NT treatments, the temperature amplitude significantly affected the early fecundity (*χ*^2^ = 18.628, *n* = 123, *p* < 0.001; *χ*^2^ = 56.515, *n =* 109, *p* < 0.001). Under the PT treatment, the temperature amplitude did not significantly impact the early fecundity (*χ*^2^ = 34.932, *n* = 103, *p* = 0.071). Under the SC treatment, compared with +/−0 °C, the early fecundity at +/−6 and +/−12 °C increased by 5.1% and 19.8%, respectively. Under the NT treatment, compared with +/−0 °C, the early fecundity at +/−6 and +/−12 °C decreased by 22.0% and 18.6%, respectively (Table S2 and Figure 5).

### 3.5. Effects of Temperature Amplitude and Insecticide on Daily Nymph Reproduction

**Figure 6:**
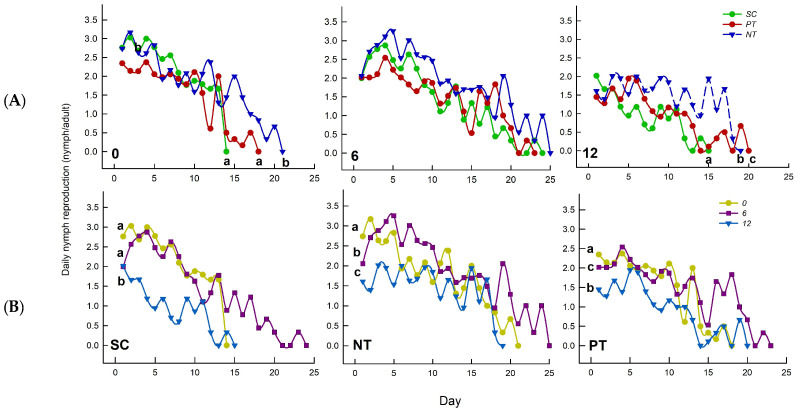
Effects of (**A**) temperature amplitude and (**B**) insecticide treatment experienced by *Sitobion avenae* nymphs on daily nymph reproduction. Different letters above the curve indicate significant differences between treatments (*p* < 0.05). The different temperature amplitudes (22 +/− 0, 22 +/− 6, and 22 +/− 12 °C) obtained are represented by 0, 6, and 12. SC, NT, and PT represent solvent control, beta-cypermethrin, and imidacloprid treatments, respectively.

Temperature amplitude (*Z* = 46.738, *n* = 27, *p* < 0.001) and insecticide (*Z* = 24.086, *n* = 27, *p* < 0.001) markedly influenced the daily nymph reproduction, but their interaction had no significant effects (*Z* = 1.209, *n* = 27, *p* = 0.342) (Table 1). At +/−0 °C, insecticides significantly influenced daily nymph reproduction (*Z* = 15.738, *n* = 9, *p* = 0.004), with SC and PT treatments showing a significantly faster decline compared to NT treatment. At +/−12 °C, insecticides markedly influenced the daily nymph reproduction (*Z* = 37.627, *n* = 9, *p* < 0.001), the order of daily nymph reproduction declined from rapid to slow with increasing age was PT, NT, and SC treatments. At +/−6 °C, insecticides significantly influenced daily nymph reproduction (*Z* = 4.635, *n* = 9, *p* = 0.061). Under SC, NT, and PT treatments, temperature amplitude (*Z* = 8.977, *n* = 9, *p* = 0.016; *Z* = 32.498, *n* = 9, *p* < 0.001; *Z* = 67.289, *n* = 9, *p* < 0.001) had significant effects on the daily nymph reproduction. Under SC and NT treatment, the order of daily nymph reproduction declined from rapid to slow with increasing age was +/−0, +/−6, and +/−12 °C. Under PT treatment, the order of daily nymph reproduction declined from rapid to slow with increasing age was +/−0, +/−12, and +/−6 °C (Figure 6).

### 3.6. Effects of Temperature Amplitude and Insecticide on the Intrinsic Rate of Increase

**Figure 7:**
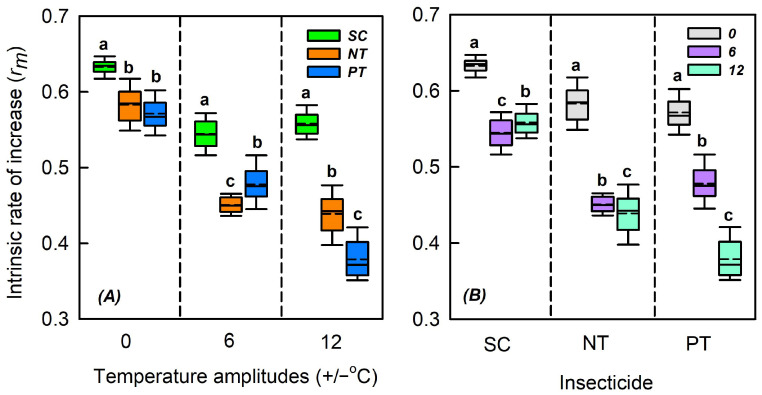
Box plot of effects of (**A**) temperature amplitude and (**B**) insecticide treatment experienced by *Sitobion avenae* nymphs on the intrinsic rate of increase. The upper and lower boundaries of the box indicate the 75th and 25th percentiles of the dataset, respectively. The black horizontal line and short dashed line within the box represent the median and mean values, respectively. Error bars represent the standard deviation. Different letters above each box indicate significant differences (*p* < 0.05) among the treatments. Different temperature amplitudes are represented by 0, 6, and 12. SC, NT, and PT represent solvent control, beta-cypermethrin, and imidacloprid treatments, respectively.

Temperature amplitude (*χ*^2^ = 6015.801, *n* = 891, *p* < 0.001), insecticide (*χ*^2^ = 3597.393, *n* = 891, *p* < 0.001), and their interaction (*χ*^2^ = 986.620, *n* = 891, *p* < 0.001) markedly influenced the intrinsic rate of increase (Table 2). At +/−0, +/−6, and +/−12 °C, insecticides markedly influenced the intrinsic rate of increase (*χ*^2^ = 17.068, *n* = 297, *p* < 0.001; *χ*^2^ = 223.174, *n* = 297, *p* < 0.001; *χ*^2^ = 245.687, *n* = 297, *p* < 0.001). At +/−0 °C, compared with the SC treatment, the intrinsic rates of increase in NT and PT treatments decreased by 0.062 and 0.050, respectively. At +/−6 °C, compared with the SC treatment, the intrinsic rate of increase in NT and PT treatments decreased by 0.067 and 0.094, respectively. At +/−12 °C, compared with the SC treatment, the intrinsic rates of increase in NT and PT treatments decreased by 0.179 and 0.119, respectively. Under PT, NT, and SC treatments, the temperature amplitude markedly influenced the intrinsic rate of increase (*χ*^2^ = 206.046, *n* = 297, *p* < 0.001; *χ*^2^ = 262.202, *n* = 297, *p* < 0.001; *χ*^2^ = 201.571, *n* = 297, *p* < 0.001). Under the SC treatment, compared with +/−0 °C, the intrinsic rates of increase at +/−6 and +/−12 °C reduced by 0.088 and 0.075, respectively. Under the NT treatment, compared with +/−0 °C, the intrinsic rates of increase at +/−6 and +/−12 °C reduced by 0.093 and 0.075, respectively. Under the PT treatment, compared with +/−0 °C, the intrinsic rates of increase at +/−6 and +/−12 °C reduced by 0.132 and 0.144, respectively (Table S2 and Figure 7).

## 4. Discussion

### 4.1. Effects of Insecticides

At the single low-dose insecticide, the PT treatment significantly reduced the longevity, fecundity, and intrinsic rate of increase at the constant temperature (+/−0 °C), and the NT treatment significantly reduced the daily nymph reproduction and intrinsic rate of increase. Low-dose insecticides can decrease the life history traits of insects [28]. We also discovered that compared with SC and PT treatments, adults had the least longevity, fecundity, and intrinsic rate of increase under the NT treatment, which was the same as the LC_10_. This may be because temperature coefficients vary between different insecticides [29]. Previous research has revealed that imidacloprid is considered a PT coefficient insecticide [30], whereas beta-cypermethrin is considered an NT coefficient insecticide [31]. In this experiment, the sensitivity of the response of *S. avenae* to PT was greater at a constant temperature of 22 °C.

### 4.2. Effects of Temperature Amplitudes

At the single temperature amplitude, the exposure of the nymph to the wide amplitude (+/−12 °C) had a significant delay effect on the adult. This result is not consistent with the “modular life cycle” hypothesis, which proposes that the ecological or physiological effects arising from external stresses in one stage are not transmitted to the next stage in the continuous life cycle [32]. In the present study, the wide amplitude (+/−12 °C) significantly decreased the fecundity, daily nymph reproduction, and the intrinsic rate of increase as well as significantly enhanced the early fecundity. This could be attributed to the resource allocation resulting from the daily high temperatures (22 +/− 12 °C with a daily maximum of 34 °C). To improve the thermal tolerance of the adult and prolong its longevity, thereby reducing reproductive energy [33], the aphid may also undergo an incomplete metamorphosis. This is because insects that undergo a complete metamorphosis pass through the pupal stage (the developmental stage preceding metamorphosis or eclosion), which allows for restoration and reconstruction of their morphology and physiological metabolism, thereby reducing the impact of the thermal stress on subsequent life stages [34]. We discovered that early fecundity at the wide amplitude (+/−12 °C) was markedly greater compared to both a constant temperature (+/−0 °C) and a medium amplitude (+/−6 °C). Environmental stress can prompt adults to reproduce earlier to mitigate adverse effects on later life stages [35]. In our study, a daily peak temperature of 34 °C with a wide fluctuation range (+/−12 °C) significantly enhanced early fecundity, which may indicate a thermal stress response. This response was generated by adverse external conditions, resulting in accelerated offspring production. By doing so, the adults were able to achieve maximum fitness and provide a means for their offspring to escape the emerging adverse environmental conditions [36].

The median amplitude (+/−6 °C) experienced by the nymph did not result in a significant delay in the survival, longevity, fecundity, or daily nymph reproduction; however, it did significantly decrease the intrinsic rate of increase. At the medium amplitude (+/−6 °C), the daily maximum temperature (28 °C) remains below the threshold at which severe injury occurs (30 °C) [37] and suitable temperatures at night (16–22 °C) are longer. Under these favorable conditions, *S. avenae* can repair most of the damage caused by mild thermal injury [38] and greatly reduce the negative delay effects on the adult. This result is in contrast to the significant negative delay effects at the wide amplitude (+/−12 °C). Therefore, the impact of a suitable night temperature on the recovery of the degree of thermal stress is limited. Furthermore, the interaction between the thermal damage caused by the daily maximum temperature and the degree of recovery of the suitable temperature at night may result in the delay effect of the temperature amplitude.

### 4.3. Interaction Effects of Insecticides and Temperature Amplitudes

The interaction between insecticide and temperature amplitude had a significant delay effect on the adult. Climate change-related environmental factors can alter insect sensitivity to insecticides, a phenomenon termed “CITS” [39]. Our results revealed that the interaction between insecticide and temperature amplitude experienced by the nymph affected longevity, survival, early fecundity, fecundity, daily nymph reproduction, and the intrinsic rate of increase. This demonstrates that temperature amplitude significantly affects the sensitivity of adults to insecticides. It can be concluded that the diurnal temperature amplitude is strongly correlated with the effect of insecticides on insects.

We observed that insecticides reduced the negative delay effect caused by the wide amplitude (+/−12 °C) on adults and, in some cases, even reversed the effect. Under the PT treatment, the fecundity and early fecundity rates at the wide amplitude (+/−12 °C) were similar to those at the constant temperature (+/−0 °C); however, the longevity and survival were significantly elevated compared to the constant temperature (+/−0 °C). Under the NT treatment, the longevity and fecundity were similar to those at the constant temperature (+/−0 °C); however, the survival was significantly higher than that of the constant temperature (+/−0 °C). The wide amplitude (+/−12 °C) could significantly reduce the survival, longevity, and fecundity of *S. avenae* [40]. This conclusion is inconsistent with our results. This may be because of the synergistic effect of insecticides and temperature stress. In the experiment, the daily maximum temperature briefly peaked at 34 °C at the wide amplitude (+/−12 °C), surpassing the developmental threshold for *S. avenae*, which is 30 °C. When *S. avenae* exhibited resistance to this temperature stress, it required a lot of energy to increase the synthesis of heat resistance metabolites. This involved inducing the production of protective substances, such as sugars, mannitol, and polyhydroxy compounds, heat-shock proteins, and sorbitol [41], as well as improving the stability of the membrane structure by increasing the proportion of saturated fatty acids and cholesterol [42]. At the same time, these substances may exhibit a synergistic effect in response to additional exogenous pressure [43], thereby reducing the negative effects of insecticides on *S. avenae*.

Furthermore, we discovered that the insecticide and the medium amplitude (+/−6 °C) interaction had a positive effect on adult phenotypes. The results revealed that under both NT and PT treatments, fecundity, longevity, and survival at the medium amplitude (+/−6 °C) were elevated compared to the constant temperature (+/−0 °C). Under constant temperature, the survival rate of *S. avenae* was 81–90% [44] at 14–25 °C and 92–97% [45] and 88–96% [46] at 15–25 °C. Therefore, 25 °C was the optimum temperature for S. avenae development. High cholinesterase activity was observed under the optimal temperature [47]. In our study, the average temperature (22 °C) closely matched the optimal temperature of *S. avenae*. The increased cholinesterase activity reduced the sensitivity of *S. avenae* to insecticides. In addition, the medium amplitude (+/−6 °C) significantly improved the phenotype of *S. avenae* [40]. Finally, the positive effect of NT treatment on survival, longevity, and fecundity was greater than that of PT treatment at the medium amplitude (+/−6 °C). Beta-cypermethrin (PT) is negatively linked to temperature, and its insecticide sensitivity decreases with an increase in temperature [48]. Conversely, imidacloprid (NT) is a positive temperature-effect insecticide. In conclusion, daily maximum temperature (28 °C in 22 +/− 6 °C) significantly reduced the sensitivity of beta-cypermethrin to the adult phenotype compared with imidacloprid.

## 5. Conclusions

Through examining the delayed impact of diurnal temperature fluctuations and insecticides on insects, we established that diurnal temperature amplitude is a crucial environmental factor influencing insecticide toxicity. First, we confirmed that the toxic effect of insecticides measured at constant temperature in the past completely ignored the effect of the temperature amplitude on organisms. Second, we observed that the temperature amplitude experienced by the nymph significantly affected the sensitivity of the adult to insecticides. This result further reinforces the CITS theory. Finally, we discovered that insecticides with the PT coefficient significantly reduced the negative delaying impact of temperature amplitude on adults, whereas insecticides with the NT coefficient revealed a positive effect on the adult. The interaction between the temperature coefficient of insecticide and temperature amplitude is very important for the accuracy of insecticide safety assessment in the context of global climate change [49]. In our study, we have solely demonstrated the phenotypic changes in organisms resulting from insecticide temperature properties and temperature fluctuations. Future research should further explore the physiological and molecular mechanisms underlying these interactions to improve pest management strategies under climate change scenarios.

## Figures and Tables

**Table 1:** Statistical results of the temperature amplitude, insecticide, and their interaction effects on the survivorship and daily nymph reproduction of *Sitobion avenae*.

Trait	Source	Z	df	*p*
Survivorship	Temperature amplitudes (TA)	5.226	2	0.073
	Insecticide treatments (IT)	14.027	2	<0.001
	TA × IT	14.563	4	0.006
Daily nymph reproduction	Temperature amplitudes (TA)	46.738	2	<0.001
	Insecticide treatments (IT)	24.086	2	<0.001
	TA × IT	1.209	4	0.342

**Table 2:** Statistical results of the temperature amplitude, insecticide, and their interaction effects on different traits of *Sitobion avenae*.

Trait	Source	*χ* ^2^	df	*p*
Longevity	Temperature amplitudes (TA)	36.353	2	<0.001
	Insecticide treatments (IT)	42.078	2	<0.001
	TA × IT	26.739	4	<0.001
Fecundity	Temperature amplitudes (TA)	68.830	2	<0.001
	Insecticide treatments (IT)	74.899	2	<0.001
	TA × IT	42.621	4	<0.001
Early fecundity	Temperature amplitudes (TA)	15.362	2	<0.001
	Insecticide treatments (IT)	42.405	2	<0.001
	TA × IT	40.831	4	<0.001
Intrinsic rate of increase (*r*_m_)	Temperature amplitudes (TA)	6015.801	2	<0.001
	Insecticide treatments (IT)	3597.393	2	<0.001
	TA × IT	986.620	4	<0.001

## Data Availability

Data used in this study are publicly archived, accessed on 10 September 2025 and were made available to editors and reviewers at the time of submission: https://doi.org/10.6084/m9.figshare.28267640 (accessed on 24 October 2025).

## Supplementary Materials

The following supporting information can be downloaded at https://www.mdpi.com/article/10.3390/biology14111543/s1, Figure S1: The (A) daily diurnal temperature fluctuations, (B) diurnal mean temperatures, maximum temperature and minimum temperature (C) temperature amplitudes of wheat fields in May 2024; Figure S2: The (A) Hourly air relative humidity (B) the mean air relative humidity in May 2024; Figure S3: (A) Target and (B) recorded temperatures with different temperature amplitudes in different climate chambers for 5 consecutive days; Table S1: Target and actual recorded temperatures with different temperature amplitudes around 22 °C in climate chambers; Table S2: Results (mean ± standard deviation) of the temperature amplitude, insecticide and their interaction on different traits of *Sitobion avenae*.
