# Supplementary material for: Aphid Nymphs Experiencing Diurnal Temperature Fluctuation Alter the Toxicity of Adults Depending on the Role of the Insecticide Temperature Coefficients
Source: Biology (Basel). 2025 Nov 3;14(11):1543. doi: 10.3390/biology14111543 (PMC12650139; doi:10.3390/biology14111543)
Supplement: Supplementary file 1 [file biology-14-01543-s001.zip › biology-3904097-supplementary.pdf]

Supporting Information

Table S1 Target and actual recorded temperatures with different temperature amplitudes around 22 °C in climate chambers

Target temperature amplitudes (+/-°C)	Recorded temperature (mean ± SD)	
	Average temperature (°C)	Temperature amplitudes (°C)
0	22.39 ± 0.13	0.89 ± 0.20
6	21.21 ± 0.26	6.89 ± 0.15
12	21.85 ± 0.20	11.93 ± 0.28

Table S2 Results (mean ± standard deviation) of the temperature amplitude, insecticide and their interaction on different traits of Sitobion avenae

Temperature amplitude (+/- °C)	Insecticide treatment	Longevity (days)	Fecundity (nymphs/adult)	Early fecundity (%)	Intrinsic rate of increase (r_m)
0	PT	8.8 ± 4.3	15.5 ± 8.1	51.1 ± 24.7	0.583 ± 0.027
0	NT	11.1 ± 5.2	22.0 ± 11.4	45.1 ± 18.1	0.571 ± 0.025
0	SC	11.4 ± 3.1	23.8 ± 8.1	39.2 ± 14.6	0.633 ± 0.011
6	PT	12.7 ± 6.1	19.6 ± 10.7	44.3 ± 23.1	0.451 ± 0.011
6	NT	18.7 ± 5.1	37.2 ± 13.0	40.2 ± 22.7	0.478 ± 0.024
6	SC	11.2 ± 5.4	21.0 ± 11.7	23.1 ± 10.9	0.545 ± 0.022
12	PT	11.1 ± 6.0	12.3 ± 5.8	59.0 ± 21.9	0.439 ± 0.028
12	NT	13.9 ± 6.5	21.6 ± 9.4	44.4 ± 24.0	0.379 ± 0.027
12	SC	9.7 ± 2.9	10.0 ± 4.8	26.5 ± 10.3	0.558 ± 0.019

Note: PT, NT and SC stand for imidacloprid, beta-cypermethrin and solvent control, respectively.

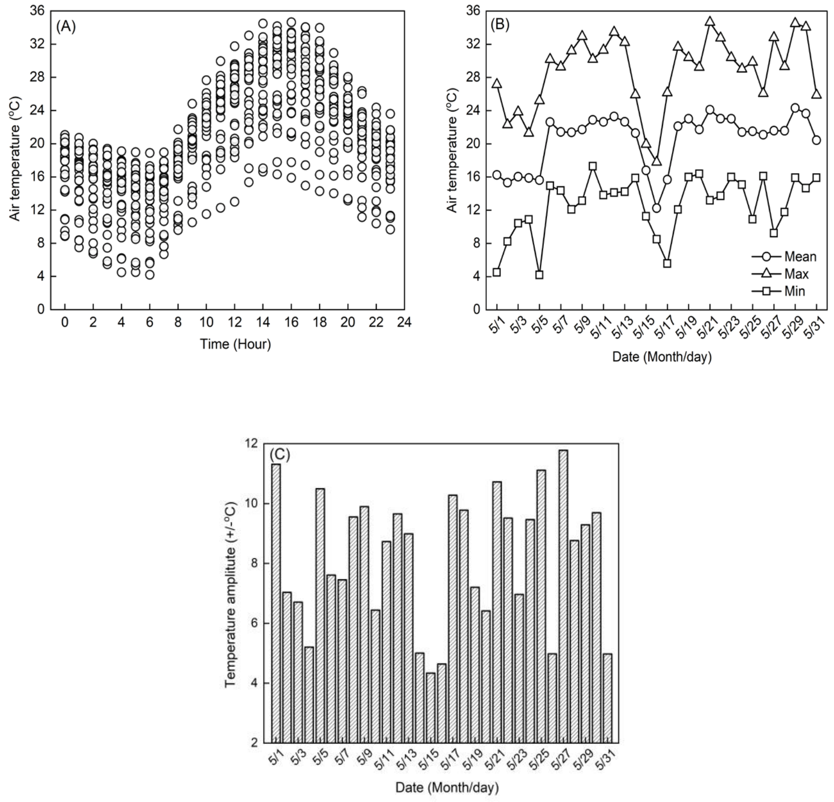

Figure S1. The (A) daily diurnal temperature fluctuations, (B) diurnal mean temperatures, maximum temperature and minimum temperature (C) temperature amplitudes of wheat fields in May 2024 .

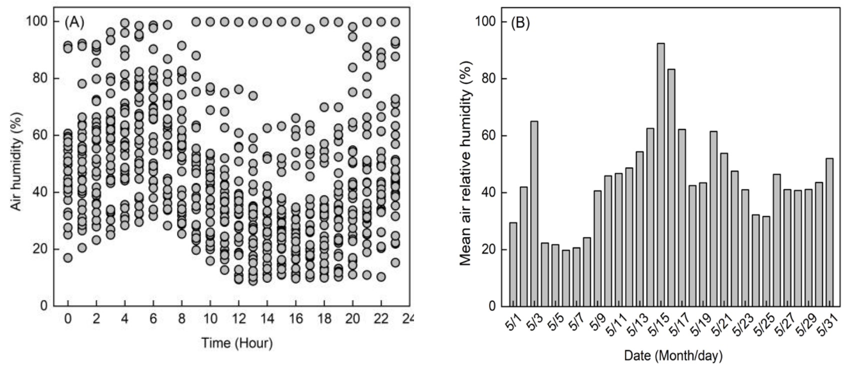

Figure S2. The (A) Hourly air relative humidity (B) the mean air relative humidity in May 2024.

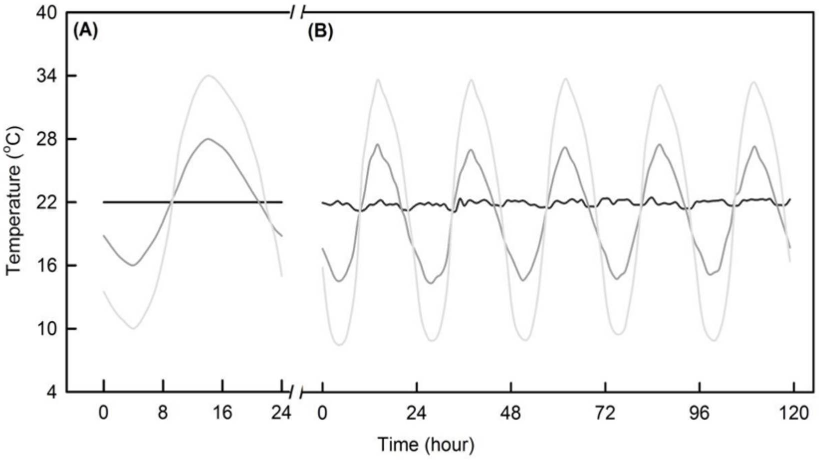

Figure S3. (A) Target and (B) recorded temperatures with different temperature amplitudes in different climate chambers for 5 consecutive days.
